# Preoperative White Matter Disease Severity Predicts Intraoperative Cerebral Desaturation During Carotid Endarterectomy

**DOI:** 10.3390/diagnostics16193212

**Published:** 2026-10-03

**Authors:** Márk Matus, Ágnes Dóra Sándor, Olaf Aleksander Ziółkiewicz, Melissa Pawin-On Brinning, Andrea Varga, Péter Sótonyi, Rita Magyar-Stang, Zsuzsanna Mihály

**Affiliations:** 1 Department of Vascular and Endovascular Surgery, Heart and Vascular Center, Semmelweis University, 1122 Budapest, Hungary; markmatusmd@gmail.com (M.M.); ziolkiewiczolaf@gmail.com (O.A.Z.);; 2 Department of Anesthesiology and Intensive Therapy, Semmelweis University, 1082 Budapest, Hungary; 3 Cardiovascular Center, Semmelweis University, 1122 Budapest, Hungary; 4 Department of Neurology, Semmelweis University, 1083 Budapest, Hungary

**Keywords:** carotid endarterectomy, intraoperative desaturation, neuromonitoring, Fazekas score, stroke risk stratification

## Abstract

**Objective**: Carotid endarterectomy carries the risk of cerebral ischemia during carotid clamping. Traditional risk stratification relies on anatomic factors such as contralateral stenosis, yet these predictors demonstrate limited sensitivity for intraoperative hypoperfusion. White matter hyperintensities (WMH), quantified by the Fazekas scale, reflect chronic cerebral small vessel disease and impaired cerebrovascular reactivity. Whether baseline WMH burden predicts intraoperative cerebral oxygen desaturation during carotid clamping remains unknown. **Methods**: This prospective, single-center cohort study enrolled patients undergoing carotid endarterectomy with continuous bilateral near-infrared spectroscopy (NIRS) monitoring. The primary outcome was the maximum contralateral regional cerebral oxygen saturation (rSO_2_) decrease within 2 min after carotid clamp placement compared with the pre-clamp baseline. The primary predictor was the contralateral hemisphere Fazekas score assessed on preoperative brain MRI. Multivariable linear regression adjusted for age, contralateral carotid stenosis, clamping time, and shunt use. **Results**: Of 173 patients screened, 58 with complete data were included in the final analysis. Th average age was 69. 2 ± 7 years, 37.9% were female, significant contralateral stenosis was present in four cases, and the average exclusion time was 30.1 ± 9.7 min. In multivariable regression, a higher contralateral Fazekas score was independently associated with greater contralateral rSO_2_ desaturation during carotid clamping (unstandardized β = 0.008, standardized β = 0.287, *p* = 0.035). The association remained statistically significant after adjustment for all covariates. The overall model explained 8.8% of variance in contralateral desaturation (R^2^ = 0.088). **Conclusions**: Contralateral white matter disease severity, quantified by Fazekas score, was independently associated with greater contralateral cerebral oxygen desaturation during carotid clamping. This finding supports the concept that baseline microvascular disease modifies acute ischemic tolerance independent of large-vessel anatomy. Preoperative Fazekas scoring may enhance risk stratification and inform individualized neuromonitoring strategies during carotid endarterectomy.

## 1. Introduction

Carotid endarterectomy (CEA) remains a cornerstone intervention for stroke prevention in patients with significant carotid artery stenosis. Yet, the procedure itself carries an inherent risk (5–20%) of perioperative cerebral ischemia during carotid clamping [1,2]. The transient interruption of ipsilateral cerebral blood flow necessitates adequate collateral perfusion and intact cerebrovascular compensatory mechanisms to maintain cerebral oxygenation [3,4]. Traditional risk stratification has focused predominantly on anatomic factors such as contralateral carotid stenosis and Circle of Willis (CoW) patency. Yet, these structural predictors alone demonstrate limited sensitivity for identifying patients who will develop intraoperative cerebral hypoperfusion [5,6]. Building on these concepts, emerging non-invasive cerebrovascular assessment techniques—such as near-infrared spectroscopy (NIRS)—offer functional insight into cerebrovascular reserve and collateral capacity that extends beyond static anatomic evaluation of the CoW. Integrating such functional perfusion assessment with intraoperative neuromonitoring may improve the prediction of hemodynamic instability during carotid clamping and enable timely corrective interventions to reduce the risk of perioperative cerebral ischemia [4].

Emerging evidence suggests that baseline cerebral microvascular integrity may be a critical, yet underappreciated, determinant of ischemic tolerance during acute hemodynamic stress [7,8,9]. White matter hyperintensities (WMH), quantified clinically using the Fazekas visual grading scale, represent a radiological marker of chronic cerebral small vessel disease and are associated with impaired cerebrovascular reserve [4,7]. Critically, reduced cerebrovascular reserve has been shown to predict periinterventional ischemic lesions during carotid revascularization, with patients developing new infarcts demonstrating 32.5% greater reduction in cerebrovascular reactivity compared to 9.2% in those without complications [10].

From a clinical perspective, white matter hyperintensity burden may provide complementary information to conventional large-vessel and collateral assessment. The Fazekas scale is a practical visual grading method that can be applied to routinely acquired preoperative FLAIR MRI without requiring additional imaging sequences or specialized post-processing. Unlike carotid stenosis severity or Circle of Willis configuration, which primarily characterize macrovascular anatomy, WMH burden reflects the cumulative effects of chronic small-vessel injury, endothelial dysfunction, and impaired cerebral autoregulation [3,4,7]. These abnormalities may reduce the capacity of the cerebral microcirculation to compensate for the abrupt decrease in perfusion pressure occurring during carotid cross-clamping. Consequently, patients with similar carotid anatomy may demonstrate substantially different cerebral oxygenation responses due to the differences in underlying microvascular reserve. Incorporating a readily available marker of cerebral small-vessel disease into preoperative assessment could therefore improve identification of patients who are particularly vulnerable to intraoperative hypoperfusion. However, the relationship between Fazekas-defined white matter disease severity and acute NIRS-detected cerebral desaturation during carotid endarterectomy has not been adequately characterized.

Near-infrared spectroscopy (NIRS) provides continuous, non-invasive monitoring of regional cerebral oxygen saturation (rSO_2_) during CEA and has demonstrated utility in detecting intraoperative cerebral ischemia [5,11,12]. A relative decrease in rSO_2_ of ≥9% demonstrates 91–95% sensitivity and 81–82% specificity for predicting ischemic symptoms during carotid clamping under local anesthesia, outperforming stump pressure measurements [5,12]. However, NIRS exhibits high negative predictive value (99%) but limited positive predictive value, and its performance varies with baseline cerebral physiology, anesthesia type, and extracranial signal contamination [13,14]. Importantly, the existing NIRS-CEA literature has focused primarily on threshold validation against clinical endpoints, with limited investigation of patient-specific physiological factors that modify cerebral oxygen desaturation responses.

Despite robust evidence linking WMH burden to impaired cerebrovascular reactivity and reduced ischemic tolerance in stroke populations, no prior studies have directly examined whether white matter disease severity predicts intraoperative cerebral oxygen desaturation during carotid clamping. This knowledge gap is clinically significant: if WMH burden independently predicts NIRS-detected desaturation, then it would support preoperative brain imaging as a tool for individualized risk stratification and monitoring strategies during CEA. We hypothesized that Fazekas scores would be independently associated with higher cerebral oxygen desaturation during carotid clamping. To test this hypothesis, we conducted a prospective cohort study using continuous bilateral NIRS monitoring in patients undergoing CEA.

## 2. Methods

### 2.1. Study Population and Design

This was a prospective, single-center cohort study conducted at the Department of Vascular and Endovascular Surgery, Semmelweis University, Budapest. The study was registered at ClinicalTrials.gov (NCT03840265 12 February 2019) and conducted in accordance with the Declaration of Helsinki. Institutional review board approval was obtained, and all patients provided written informed consent prior to enrollment. A total of 173 consecutive patients were screened, of whom a complete-case subset was included in the final regression analysis. All patients underwent carotid endarterectomy under general anesthesia through a longitudinal incision along the medial border of the sternocleidomastoid muscle, followed by exposure of the carotid bifurcation. In most patients, eversion endarterectomy was performed by trans-section of the carotid bifurcation, eversion of the arterial wall with plaque removal, and subsequent end-to-end reanastomosis. Patients requiring intraoperative shunting with a Pruitt–Inahara shunt underwent conventional endarterectomy through a longitudinal arteriotomy extending from the common carotid artery to the internal carotid artery, followed by bovine patch angioplasty.

### 2.2. Inclusion and Exclusion Criteria

Patients were included in the analysis based on the prospective study description (below Table 1) only if complete, non-missing numeric data were available for all variables required for the multivariable regression model. Specifically, inclusion required availability of the following variables: contralateral cerebral oxygen desaturation during carotid clamping (defined below), contralateral Fazekas scale score on preoperative MR skull scan, age, contralateral carotid stenosis status, clamping time, and shunt use. Patients with missing data in any of these variables were excluded from the regression analysis. After applying these criteria and excluding patients without pre- and postoperative cranial MRI, 58 patients with complete data were included in the final analysis.

### 2.3. Near-Infrared Spectroscopy Monitoring

The rSO_2_ measurement was performed using INVOS ^®^ 4000 cerebral oximeter (Somanetics, Troy, MI, USA). Two oximeter sensors were placed on the forehead, and bilateral rSO_2_ measurements were recorded continually throughout the operation. The numerical rSO_2_ readings were recorded at 5 or 10 s intervals and stored on a computer for off-line data analysis in postoperative time period. Bilateral cerebral oximetry was performed continuously throughout the procedure using near-infrared spectroscopy (NIRS). Regional cerebral oxygen saturation (rSO_2_) was recorded on both the ipsilateral and contralateral sides. The dependent variable was defined as the maximum decrease in contralateral rSO_2_ (%) occurring within 2 min after carotid clamp placement compared with baseline values obtained during the 2 min immediately preceding clamping. This metric was selected a priori based on prior methodological work and internal validation demonstrating its sensitivity to cerebral desaturation during carotid clamping [5].

### 2.4. Assessment of White Matter Disease

The MRI examinations were performed on a 1.5T scanner (Magnetom Aera 1.5, Siemens Healthcare, Erlangen, Germany). The preoperative MRI protocol included diffusion MRI (DTI), 3D T1-weighted gradient echo, 3D fluid-attenuated inversion recovery (FLAIR), and susceptibility-weighted imaging (SWI) sequences. Whole-brain coverage was obtained with 5 mm-thick axial slices and a gap of 1.5 mm. The total acquisition time was 5:30–6:30 min. The postoperative scans were performed with a shorter protocol including DTI, 3D T1-weighted and SWI sequences. White matter small-vessel disease severity was assessed using the Fazekas visual grading scale applied to preoperative brain magnetic resonance imaging. The Fazekas scale grades white matter hyperintensities from 0 to 3, with higher scores indicating greater severity of white matter disease in both hemispheres.

**Figure 1:**
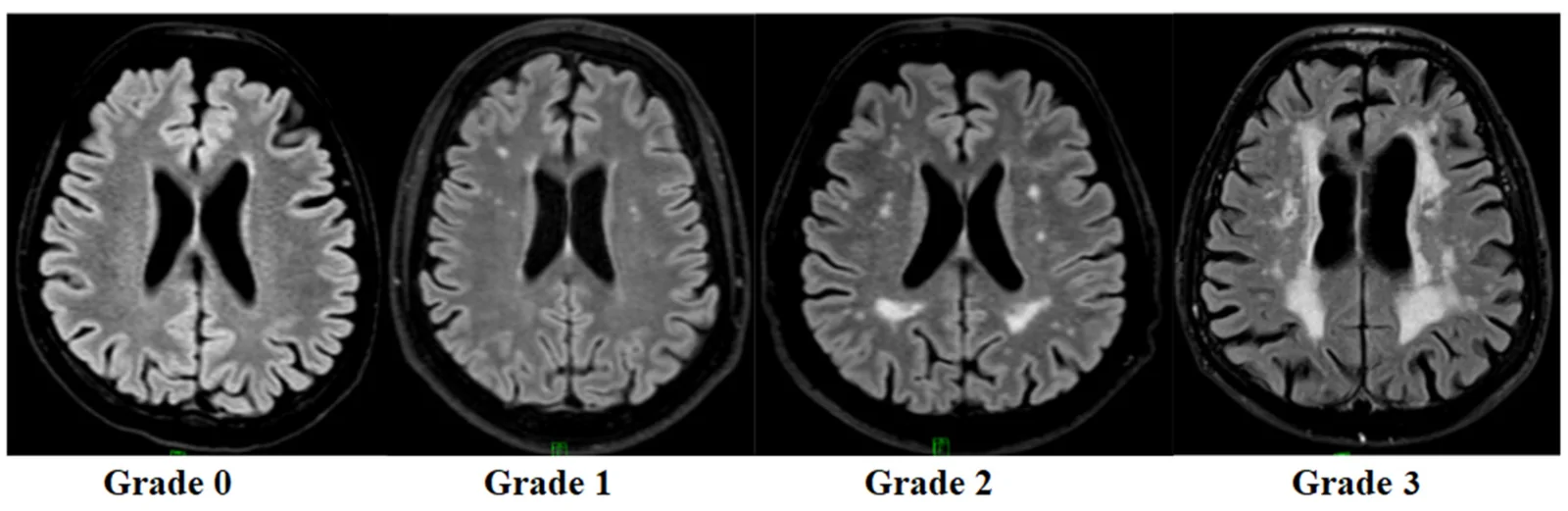
Representative axial fluid-attenuated inversion recovery magnetic resonance images illustrating increasing white matter hyperintensity severity according to the Fazekas scale. Images demonstrate Fazekas grade 0, no white matter hyperintensities; grade 1, punctate foci; grade 2, beginning confluence of lesions; and grade 3, large confluent areas of white matter hyperintensity.

Representative axial FLAIR images illustrating the Fazekas grading system are shown in Figure 1.

### 2.5. Statistical Analysis

The association between contralateral Fazekas scale score and maximum contralateral rSO_2_ drop was evaluated using multivariable ordinary least squares (OLS) linear regression. The Fazekas score was specified as the primary predictor, with age, contralateral stenosis status, clamping time, and shunt use entered simultaneously as covariates to adjust for potential confounding. The regression model was defined as:

Max rSO_2_ drop = β0 + β1(Fazekas) + β2(Age) + β3(Contralateral stenosis) + β4(Clamping time) + β5(Shunt) + ε

where ε represents the residual error term.

The following covariates were included in the multivariable model based on their established or plausible influence on cerebral perfusion during carotid clamping: age (years, continuous variable); contralateral carotid stenosis (binary variable: 0 = nonsignificant stenosis < 50%, 1 = significant stenosis ≥ 50% or occlusion); clamping time (minutes, continuous variable); and shunt use (binary variable: 0 = no shunt, 1 = shunt used). Given the limited complete-case sample size, a relatively simple multivariable model was used to minimize the risk of overfitting. Age, contralateral carotid stenosis, clamping time, and shunt use were selected a priori as a clinically and physiologically plausible determinants of cerebral oxygenation during carotid clamping.

All statistical analyses were performed using Python version 3.11 (Python Software Foundation, Wilmington, DE, USA), with pandas version 2.2 for data management, statsmodels version 0.14 for regression modeling, and scipy version 1.11 for statistical computations. Standard assumptions of linear regression, including linearity, homoscedasticity, and normality of residuals, were assessed using visual diagnostic methods. Results are reported as unstandardized regression coefficients (β) with 95% confidence intervals, standardized regression coefficients, and corresponding *p*-values. Model explanatory power was quantified using the coefficient of determination (R^2^). A two-sided *p*-value < 0.05 was considered statistically significant. To quantify the unique contribution of individual predictors within the multivariable model, partial R^2^ values were calculated.

## 3. Results

### 3.1. Study Population

Of the 173 patients screened, 58 patients (33.5%) met the inclusion criteria with complete data available for all variables required for the multivariable regression analysis. The remaining patients were excluded due to missing values in one or more of the following variables: maximum regional cerebral oxygen saturation (rSO_2_) drop during carotid clamping, Fazekas scale score, age, contralateral carotid stenosis status, clamping time, or shunt use. Baseline demographic and clinical characteristics of the study cohort are summarized in Table 2.

### 3.2. Preop MR Findings

Preoperative brain MRI was available for all patients included in the matched analysis cohort (n = 58). Fazekas scores were assessed separately for the ipsilateral and contralateral hemispheres. On the ipsilateral side, Fazekas grade 0 was present in 17 patients (29.3%), grade 1 in 23 patients (39.7%), grade 2 in 14 patients (24.1%), and grade 3 in 4 patients (6.9%). On the contralateral side, Fazekas grade 0 was present in 17 patients (29.3%), grade 1 in 20 patients (34.5%), grade 2 in 16 patients (27.6%), and grade 3 in 5 patients (8.6%). The median (IQR) Fazekas score was 1 (0–2) on both the ipsilateral and contralateral sides. These descriptive MRI findings were derived from the same matched patient cohort used for the regression analyses.

### 3.3. Cerebral Desaturation During Carotid Clamping

All included patients underwent continuous bilateral near-infrared spectroscopy monitoring during carotid endarterectomy. The primary outcome was the maximum decrease in rSO_2_ occurring within 2 min after carotid clamp placement compared with the 2 min pre-clamp baseline.

The mean maximal rSO_2_ decrease (2/2 min window) was −0.089 ± 0.074 on the operated side compared with −0.023 ± 0.036 on the contralateral side. Descriptive rSO_2_ analyses are shown in Figure 1 and were performed using all available measurements, whereas regression models were restricted to complete-case datasets.

**Figure 2:**
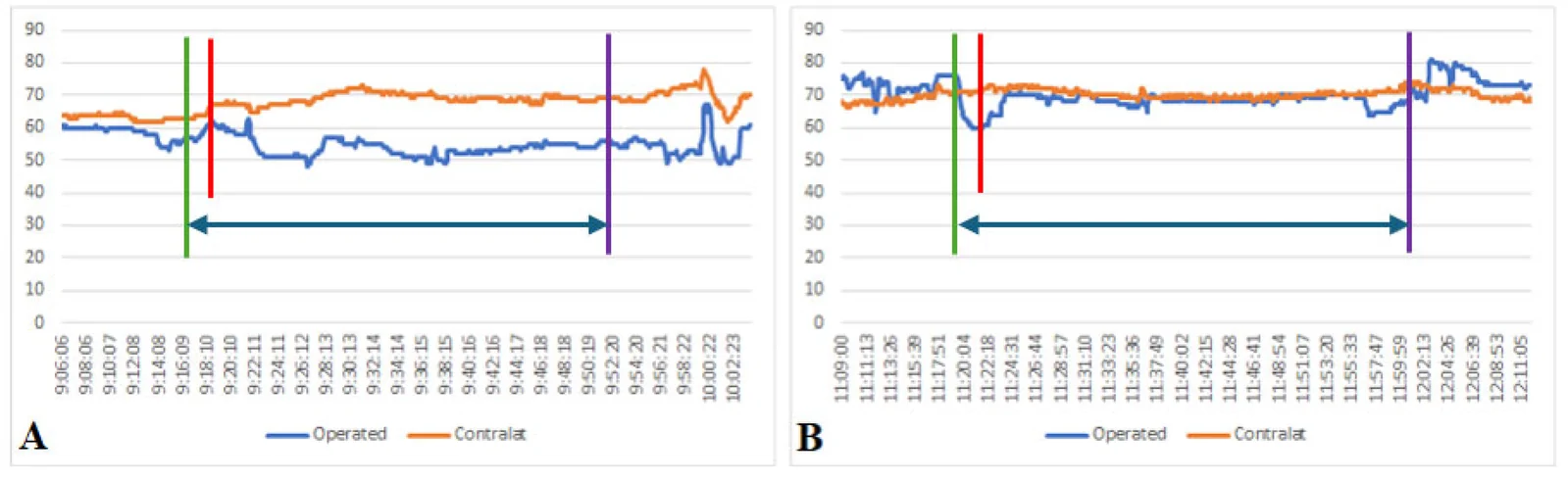
Representative intraoperative bilateral regional cerebral oxygen saturation recordings and the near-infrared spectroscopy monitoring system. Graphical illustration of changes in regional cerebral oxygen saturation (rSO_2_) over time. The green vertical line indicates carotid clamp placement, the red vertical line indicates 2 min after clamp placement corresponding to the prespecified analysis window, and the purple vertical line indicates clamp release. The blue horizontal arrow denotes the duration of carotid clamping. The *y*-axis indicates rSO_2_ (%), and the *x*-axis represents time. The blue line represents the operated side, and the orange line represents the contralateral side. Recordings begin approximately 10 min before carotid clamping and end approximately 10 min after clamp release. (**A**) Representative patient with bilateral Fazekas grade 0 with an isolated Circle of Willis system, demonstrating persistent separation between the operated-side and contralateral regional cerebral oxygen saturation trajectories. (**B**) Representative patient with bilateral Fazekas grade 3 with isolated Circle of Willis system, demonstrating bilateral different cerebral oxygenation response during carotid clamping. The recordings illustrate individual intraoperative rSO_2_ patterns and are provided as representative examples rather than as direct evidence of the association identified in the regression analysis.

Representative intraoperative NIRS recordings illustrate that patients with comparable large-vessel collateral anatomy may demonstrate different cerebral oxygenation patterns depending on the underlying burden of white matter disease. Despite contralateral Circle of Willis isolation in both representative cases, the patient with severe white matter disease demonstrated less autoregulation, whereas different contralateral cerebral oxygenation remained preserved in response to carotid clamping compared with the patient without white matter hyperintensities (Figure 2).

### 3.4. Association Between White Matter Disease and Cerebral Desaturation

In multivariable ordinary least squares linear regression analysis adjusting for age, contralateral carotid stenosis, clamping time, and shunt use, contralateral Fazekas scale score was independently associated with maximum contralateral rSO_2_ drop. The unstandardized regression coefficient for Fazekas score was β = 0.008, with a standardized coefficient of β = 0.287 (*p* = 0.035). Table 3 summarizes the results of the model.

**Figure 3:**
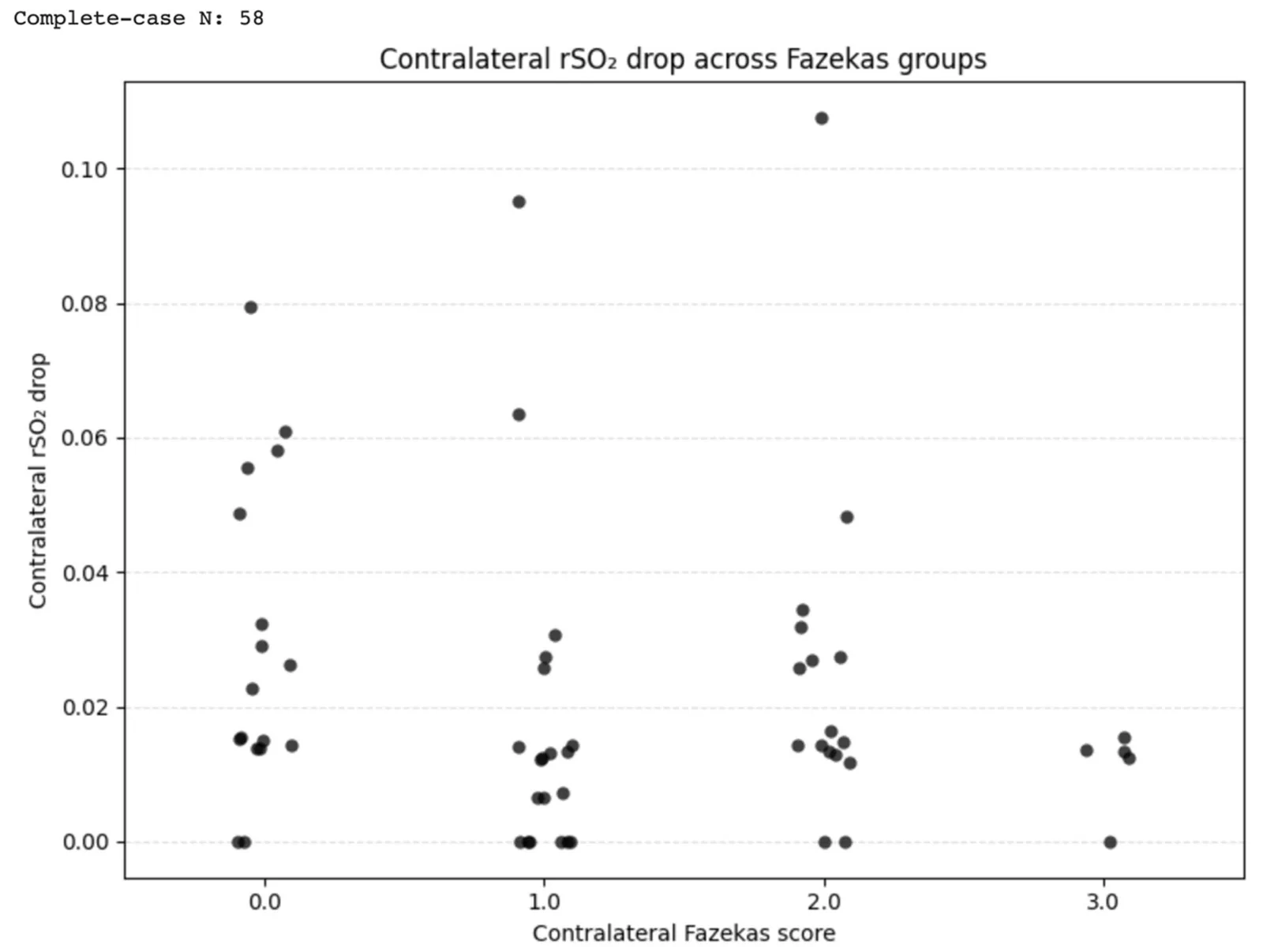
Distribution of the maximum contralateral regional cerebral oxygen saturation (rSO_2_) change during carotid clamping according to contralateral Fazekas score in the complete-case cohort (n = 58). The graphic shows the regional cerebral oxygen saturation (rSO_2_) decline in the different Fazekas grade groups. The *x*-axis represents the Fazekas grade, and the *y*-axis represents the maximum contralateral rSO_2_ change from pre-clamp baseline, whereas positive values indicate an increase expressed as a negative percentage (%) from baseline.

The overall regression model explained 8.8% of the variance in maximum contralateral rSO_2_ desaturation (R^2^ = 0.088). The association between Fazekas score and contralateral cerebral desaturation remained statistically significant after adjustment for all covariates included in the model. Descriptively, the mean ± SD maximum contralateral rSO_2_ change was −2.78 ± 2.51% for Fazekas 0 (n = 17), −1.51 ± 2.52% for Fazekas 1 (n = 20), −1.74 ± 3.13% for Fazekas 2 (n = 16), and +0.60 ± 1.19% for Fazekas 3 (n = 5), with negative values indicating decreases from the pre-clamp baseline. The distribution of these unadjusted values across Fazekas categories is shown in Figure 3. These unadjusted group-wise values did not demonstrate a monotonic gradient across increasing Fazekas categories.

Using the same complete-case cohort (N = 58), the ipsilateral (operated-side) Fazekas score was not associated with ipsilateral rSO_2_ desaturation during carotid clamping (β = −0.0011, *p* = 0.89) after adjustment for age, contralateral stenosis, clamping time, and shunt use.

## 4. Discussions

This prospective study demonstrates that contralateral white matter disease severity, quantified by Fazekas score, is independently associated with greater contralateral cerebral oxygen desaturation during carotid clamping (β = 0.008, standardized β = 0.287, *p* = 0.035), after adjustment for age, contralateral carotid stenosis, clamping time, and shunt use. To our knowledge, this is the first study to directly link preoperative white matter hyperintensity burden to intraoperative cerebral oxygen desaturation measured by NIRS during carotid endarterectomy.

### 4.1. Study Population and Complete-Case Analysis

The final analysis included 58 of 173 screened patients with complete data on all variables required for multivariable regression: contralateral rSO_2_ desaturation, contralateral Fazekas score, age, contralateral carotid stenosis, clamping time, and shunt use. This complete-case approach was necessary to ensure model validity, but it introduces important limitations. Patients with missing data may have differed systematically in baseline characteristics, disease severity, or outcomes, raising concerns about selection bias and generalizability [4,15]. The relatively small final sample size limits statistical power to detect associations of smaller magnitude and restricts our ability to include additional covariates or perform subgroup analyses. Nevertheless, the complete-case strategy ensures that all reported associations are based on uniform, high-quality data without imputation assumptions, which is particularly important given the exploratory nature of this investigation.

### 4.2. Significance of Contralateral rSO_2_ Desaturation

The primary outcome was defined as the maximum decrease in contralateral rSO_2_ occurring within 2 min after carotid clamp placement compared with the 2 min pre-clamp baseline. This metric was suspected based on prior methodological work [16]. demonstrating its sensitivity to acute cerebral desaturation during carotid clamping. The contralateral hemisphere is less exposed to surgical intervention and probably reflects the patient’s intrinsic cerebrovascular reactivity without the confounding effects of direct surgical manipulation, embolization, or variable shunt placement affecting the ipsilateral side. In addition, in most cases the chronic hypoperfusion effect caused by contralateral carotid stenosis is also absent, thus the microvasculature of the contralateral hemisphere is influenced only by the effects of classical vascular risk factors [7,16]. The contralateral hemisphere plays a critical physiological role during carotid cross-clamping through collateral recruitment via the Circle of Willis and other pathways [17,18]. Evidence from 4D phase-contrast MRI demonstrates that in patients with carotid stenosis, the contralateral internal carotid artery primarily supplies the bilateral anterior cerebral artery territory, while the ipsilateral internal carotid artery predominantly perfuses the ipsilateral middle cerebral artery territory [19]. During carotid clamping, approximately 10% of patients cannot tolerate occlusion due to inadequate collateral circulation, and a larger proportion develop low-flow states requiring augmented blood pressure to maintain perfusion through collateral channels [20]. The contralateral A1 segment exerts the strongest influence on carotid stump pressure during internal carotid artery occlusion, underscoring the dominance of anterior Circle of Willis collaterals in preserving cerebral blood flow [21]. According to a recent review article, the variability in reported effects of the Circle of Willis (CoW) may be attributed to differences in the criteria used to define complete versus incomplete configurations, variations in cohort size and imaging modalities (CTA or MRA) across studies [6], as well as the lack of high-quality studies comparing imaging findings with clinical assessments of cerebrovascular reactivity, such as functional transcranial Doppler (TCD) results. Given the lack of consensus regarding the definition and classification of CoW variants, CoW status was not included in the primary model; however, an additional sensitivity analysis incorporating CoW configuration was performed.

Our finding that contralateral Fazekas score is associated with contralateral rSO_2_ desaturation is consistent with this framework: the contralateral hemisphere reflects the patient’s baseline cerebrovascular reactivity and serves as an internal physiological control during acute hemodynamic stress. The contralateral rSO_2_ response is not intended as a direct marker of the occurrence or laterality of perioperative stroke, but rather as a potential indicator of global cerebrovascular reserve capacity impairment and vulnerability to clamp-induced hemodynamic stress. Our findings raise the hypothesis that patients with a higher Fazekas grade on the contralateral side may have a reduced capacity to compensate for ipsilateral carotid clamping; however, this hypothesis requires confirmation in larger prospective studies incorporating clinical and imaging outcomes or experimental animal studies.

Although the unadjusted group-wise values did not demonstrate a monotonic gradient across Fazekas categories, contralateral Fazekas score remained significantly associated with contralateral rSO_2_ change in the multivariable model after adjustment for the included covariates. The descriptive group-level findings should therefore be interpreted cautiously, particularly given the small number of patients with Fazekas grade 3.

The multivariable regression model adjusted for age, contralateral carotid stenosis, clamping time, and shunt use—all established or plausible determinants of cerebral perfusion during carotid clamping. The model explained 8.8% of the variance in contralateral rSO_2_ desaturation (R^2^ = 0.088), indicating that the majority of variance remains attributable to unmeasured factors. Cerebral desaturation during CEA is multifactorial, influenced by Circle of Willis anatomy, blood pressure management, anesthetic depth, hematocrit, PaCO_2_, and other variables not captured in our model [21,22,23]. Our findings that Fazekas score independently contributes to desaturation risk despite these unmeasured confounders underscore the importance of baseline microvascular integrity. The independent association between contralateral Fazekas score and contralateral rSO_2_ desaturation after adjustment for prespecified covariates supports a potential impairment of baseline microvascular responsiveness to carotid clamping. However, the variance and the presence of unmeasured physiological and anatomical factors warrant cautious interpretation of this association, a well-established determinant of white matter hyperintensity burden. White matter hyperintensity volume increases exponentially with age in both sexes, with marked interindividual variation [24]. Population-based studies demonstrate that WMH burden begins to emerge in middle age, with a critical inflection point at approximately 61 years for periventricular WMH [14]. Age-related WMH are associated with widespread macro- and microstructural changes in gray and white matter, even at Fazekas grade 1, indicating that the mere presence of WMH—not just WMH load—is an important clinical indicator of brain health [12]. The fact that Fazekas score independently predicts desaturation after controlling for age suggests that WMH burden captures pathophysiological information beyond chronological aging alone, likely reflecting individual variation in cumulative vascular injury, blood–brain barrier dysfunction, and microvascular integrity [5,13].

### 4.3. Physiological Basis: White Matter Hyperintensities and Cerebrovascular Reactivity

WMH reflects a chronic small-vessel pathology in which impaired endothelial function, altered pressure responsiveness, and disrupted neurovascular coupling coexist, leading to a globally reduced ability of the cerebral microcirculation to adapt to hemodynamic and metabolic stress. The physiological basis for the association between white matter hyperintensities and cerebral desaturation is supported by converging evidence linking WMH to impaired cerebrovascular reactivity. White matter hyperintensities represent a radiological marker of chronic cerebral small vessel disease characterized by endothelial dysfunction, arteriolosclerosis, and loss of autoregulatory capacity [13,25]. Patients with higher WMH burden demonstrate reduced cerebrovascular reactivity to hypercapnic challenge, with each 10-fold increase in WMH volume associated with a −0.0073%/mm Hg decrease in CO_2_ reactivity in normal-appearing white matter [4]. Longitudinal studies demonstrate that reduced cerebrovascular reactivity precedes the development of new WMH, with areas of low CVR showing 26.5% lower reactivity compared to contralateral regions that did not progress to WMH over one year [17]. During carotid clamping, the sudden reduction in perfusion pressure places substantial demands on the brain’s compensatory capacity: patients with preserved cerebrovascular reactivity are able to maintain cerebral blood flow through maximal vasodilation and effective collateral recruitment, whereas those with pre-existing microvascular pathology and impaired or exhausted CVR are unable to achieve an adequate compensatory response [17,19].

### 4.4. Absence of Ipsilateral Association

In patients with severe carotid stenosis, the ipsilateral hemisphere is exposed to chronic hemodynamic impairment and adaptive changes, which further increase the complexity of the relationship between WMH and acute desaturation. Evidence demonstrates that ipsilateral cerebral blood flow is already reduced in patients with carotid stenosis, with internal carotid artery flow rates significantly lower on the stenotic side (134 ± 87 versus 261 ± 95 mL/min contralaterally) [26]. Despite collateral recruitment via reversed flow in the ophthalmic and posterior communicating arteries, in some cases, ipsilateral middle cerebral artery flow remains compromised. Critically, the degree of ipsilateral perfusion impairment does not correlate linearly with stenosis severity due to variable collateral compensation, making ipsilateral perfusion metrics less reliable predictors of acute ischemic tolerance [19]. Furthermore, the ipsilateral hemisphere is subject to procedural noise during CEA—including direct surgical manipulation, embolization, and variable shunt placement—that may introduce confounding variability in rSO_2_ measurements independent of baseline WMH burden [14]. In contrast, the contralateral hemisphere remains anatomically remote from the surgical field and reflects the patient’s intrinsic cerebrovascular reserve capacity without these confounding influences. This asymmetry in measurement reliability may explain why the contralateral association is detected while the ipsilateral association is not and underscores the value of bilateral NIRS monitoring to capture patient-level physiological reserve [5].

The lack of an ipsilateral association may reflect greater peri-procedural variability on the operated side (surgical manipulation, local hemodynamic fluctuations, and shunt-related flow changes), which can introduce measurement noise and attenuate physiological relationships. In contrast, the contralateral hemisphere may provide a more stable signal of cerebrovascular vulnerability during clamping.

### 4.5. Model Performance and Unmeasured Factors

The modest explained variance (R^2^ = 0.088) reflects the complex, multifactorial nature of cerebral desaturation during CEA. Important unmeasured variables likely include anatomic completeness of the Circle of Willis, real-time blood pressure variability, anesthetic depth, hematocrit, PaCO_2_, effect of general anesthesia and individual differences in cerebrovascular reactivity [21,22,23,27]. General anesthesia can reduce cerebral metabolic demand and may thereby increase tolerance to transient reductions in cerebral perfusion, potentially attenuating the magnitude of ischemia-related cerebral oxygen desaturation observed during carotid clamping [28]. The anterior Circle of Willis dominates in preserving collateral flow during ICA occlusion, especially the contralateral A1 segment, and anatomic variations in communicating artery caliber exert strong influences on carotid stump pressure and ischemic tolerance [21]. Blood pressure management during clamping, which was not standardized or captured in our model, directly affects collateral perfusion and may modify the relationship between baseline WMH and acute desaturation [20]. Despite these unmeasured confounders, the independent contribution of Fazekas score to desaturation risk supports the hypothesis that baseline microvascular disease modifies acute ischemic tolerance independent of large-vessel anatomic factors.

### 4.6. Study Limitations

This study has several limitations. First, it was conducted at a single center and included only 58 of the 173 screened patients because the multivariable analysis required complete data for all included variables, which is possible only in case of high-quality data collection regarding all variables for all subjects. This complete-case approach may have introduced selection bias if patients with missing data differed systematically from those included in the analysis, and it limits the generalizability of the findings. Second, the relatively small sample size, together with the low number of patients with significant contralateral carotid stenosis and the infrequent use of intraoperative shunting, limited statistical power and prevented meaningful subgroup analyses. Third, the regression model explained only a modest proportion of the variability in contralateral cerebral desaturation. Important determinants of cerebral perfusion, including Circle of Willis anatomy, intraoperative blood pressure, arterial carbon dioxide levels, hematocrit, and anesthetic depth, were not available for inclusion in the model. Fourth, the Fazekas scale is an ordinal visual grading system and may be less sensitive than quantitative white matter hyperintensity volumetry. Interobserver reliability was not formally assessed in this analysis. Finally, cerebral desaturation measured by NIRS represents a physiological surrogate outcome. We did not establish whether Fazekas-associated desaturation was associated with perioperative stroke, new diffusion-weighted MRI lesions, or postoperative cognitive decline. Therefore, these findings should be considered exploratory and require external validation before Fazekas scoring can be used to guide intraoperative management.

NIRS has well-documented limitations that must be acknowledged. NIRS predominantly measures capillary oxygenation (which comprises approximately 80% of cerebral blood volume) rather than arterial oxygen delivery, and extracranial signal contamination from scalp and skull can range from 3% to 50% depending on device and patient characteristics. Furthermore, NIRS assesses cerebral oxygenation primarily in the frontal cortex, whereas the Fazekas score reflects white matter changes distributed throughout the deep and subcortical regions, which may limit the direct comparability of these measures. NIRS demonstrates high negative predictive value for cerebral ischemia but poor positive predictive value and low specificity, resulting in potential overestimation of ischemic events [23]. Systematic reviews report that NIRS sensitivity and specificity vary widely across studies, with a Δ20% threshold under local anesthesia yielding a pooled sensitivity of 70.5% and a specificity of 92.4% compared with awake neurological monitoring. These limitations underscore the importance of interpreting NIRS data in the context of other clinical and physiological parameters rather than as a standalone measure of cerebral ischemia.

### 4.7. Clinical Implications and Future Directions

Clinical implications of our findings are the following: First, preoperative Fazekas scoring may enhance risk stratification beyond anatomic assessment alone, identifying patients who require more aggressive hemodynamic management, relatively higher NIRS desaturation thresholds for shunt placement, or consideration for alternative revascularization strategies [20]. Second, our results support the use of bilateral NIRS monitoring to capture patient-level cerebrovascular reserve, rather than relying solely on ipsilateral monitoring [5]. Third, the association between WMH burden and desaturation suggests that patients with high Fazekas scores may benefit from multimodal neuromonitoring (e.g., transcranial Doppler, evoked potentials) to improve detection of ischemia, as no single monitoring modality is comprehensive [23].

Future directions should include prospective validation in larger, multicenter cohorts with complete outcome ascertainment, including postoperative MRI for diffusion-weighted lesions and neurocognitive testing [13,20,29,30,31,32]. We did not assess clinical outcomes (stroke, cognitive decline, diffusion-weighted imaging lesions), limiting our ability to determine whether Fazekas-predicted desaturation translates to patient harm [20,31]. Integration of preoperative cerebrovascular reserve testing (e.g., CO_2_ reactivity, acetazolamide challenge) with intraoperative NIRS monitoring could provide a comprehensive assessment of ischemic vulnerability [4,17,31]. Additionally, investigating whether Fazekas-guided monitoring strategies (e.g., lower shunt thresholds in high-WMH patients) improve outcomes would establish their clinical utility. Finally, machine learning approaches incorporating WMH burden, anatomic collaterals, and real-time physiological data may enable personalized risk prediction models.

## 5. Conclusions

Contralateral white matter disease severity, assessed by Fazekas score, was independently associated with greater contralateral cerebral oxygen desaturation during carotid clamping. Our finding supports the emerging paradigm that baseline cerebrovascular reserve—reflected by chronic microvascular injury—modifies acute ischemic tolerance independent of large-vessel anatomy and chronological age. The contralateral hemisphere could serve as a physiological mirror of patient-level cerebral microcirculatory reserve capacity, unconfounded by the ipsilateral hemodynamic impairment and procedural noise inherent to severe carotid stenosis and surgical manipulation. Preoperative Fazekas scoring may enhance risk stratification and inform individualized monitoring strategies during carotid endarterectomy.

## Figures and Tables

**Table 1:** Study inclusion and exclusion criteria. Abbreviations: CT, computed tomography; MRI, magnetic resonance imaging; NASCET, North American Symptomatic Carotid Endarterectomy Trial.

Assessment Category	Inclusion Criteria	Exclusion Criteria
General eligibility	Written informed consent	Withdrawal of informed consent; age < 50 years; neurological event within 15 days before surgery
Vascular surgery criteria	Significant carotid artery stenosis (≥70% according to NASCET criteria); planned carotid endarterectomy	—
Carotid CT angiography	Examination performed using a Brilliance iCT 256 scanner (Philips Healthcare, Cleveland, OH, USA)	Stage V chronic kidney disease; severe motion artifacts
Cranial MRI	Examination performed using a 1.5-T Magnetom Aera scanner (Siemens Healthcare, Erlangen, Germany)	Pacemaker implantation; severe motion artifacts

**Table 2:** Baseline demographic and clinical characteristics of the study cohort. Data are presented as mean ± SD, or n (%), as appropriate. Percentages are calculated using the total cohort as the denominator. Abbreviations: SD, standard deviation; COPD, chronic obstructive pulmonary disease; IHD, ischemic heart disease; AF, atrial fibrillation; ASA, aspirin; CRF, chronic renal failure.

Characteristic	Value
N	58
Age (years), mean ± SD	69.2 ± 7.0
Female sex, n (%)	22 (37.9%)
Smoking, n (%)	16 (28.6%)
Hypertension, n (%)	55 (94.8%)
Diabetes, n (%)	20 (34.5%)
IHD, n (%)	11 (19.0%)
AF, n (%)	2 (3.4%)
CRF, n (%)	0 (0.0%)
COPD, n (%)	8 (13.8%)
ASA, n (%)	35 (60.3%)
Clopidogrel, n (%)	27 (46.6%)
Statin, n (%)	45 (77.6%)
Shunt used, n (%)	1 (1.7%)
Significant contralateral stenosis, n (%)	4 (6.9%)
Clamping time (min), mean ± SD	30.1 ± 9.7

**Table 3:** Presenting the complete multivariable linear regression model result. Abbreviation: Sc_ score Ne: not estimable Nv: No variation. The overall regression model explained 8% of the variance in maximum contralateral rSO_2_ desaturation (R^2^ = 0.088). The association between Fazekas score and contralateral cerebral desaturation remained statistically significant after adjustment for all covariates included in the model. Using the same complete-case cohort (N = 58), the ipsilateral (operated-side) Fazekas score was not associated with ipsilateral rSO_2_ desaturation during carotid clamping (β = −0.0011, *p* = 0.89) after adjustment for age, contralateral stenosis, clamping time, and shunt use. Additional adjustment for Circle of Willis configuration did not materially alter the association between contralateral Fazekas score and maximum contralateral rSO_2_ decrease (β = 0.0082, *p* = 0.036). Circle of Willis configuration itself was not significantly associated with contralateral rSO_2_ decrease (*p* = 0.386). The extended model explained 10.1% of the variance (R^2^ = 0.101), compared with R^2^ = 0.088 in the primary model.

Predictor	Unstandardized β	95% CI	Standardized β	*p*-Value	Partial R^2^
Contralateral Fazekas sc.	0.008205	0.000611–0.015799	0.287	0.0347	0.0814
Age (years)	−0.000350	−0.001390–0.000689	−0.094	0.5017	0.0086
Contralateral stenosis	0.000646	−0.021273–0.022566	0.008	0.9531	0.0001
Clamping time (min)	0.000039	−0.000707–0.000785	0.014	0.9166	0.0002
Shunt use	Ne	Nv in N = 58	Ne	Ne	Ne

## Data Availability

The data underlying this article cannot be shared publicly because of institutional and participant confidentiality restrictions. De-identified data may be made available by the corresponding author upon reasonable request and subject to institutional approval.
